# A Cognitive Level Evaluation Method Based on a Deep Neural Network for Online Learning: From a Bloom’s Taxonomy of Cognition Objectives Perspective

**DOI:** 10.3389/fpsyg.2021.661235

**Published:** 2021-10-14

**Authors:** Yan Cheng, Yingying Cai, Haomai Chen, Zhuang Cai, Gang Wu, Jing Huang

**Affiliations:** ^1^School of Computer Information Engineering, Jiangxi Normal University, Nanchang, China; ^2^School of Computer Science, Neusoft Institute Guangdong, Foshan, China; ^3^School of Mathematics and Computer, Yuzhang Normal University, Nanchang, China

**Keywords:** online learning, cognitive level evaluation, deep neural network, interactive text, Bloom’s cognitive taxonomy

## Abstract

The evaluation of the learning process is an effective way to realize personalized online learning. Real-time evaluation of learners’ cognitive level during online learning helps to monitor learners’ cognitive state and adjust learning strategies to improve the quality of online learning. However, most of the existing cognitive level evaluation methods use manual coding or traditional machine learning methods, which are time-consuming and laborious. They cannot fully mine the implicit cognitive semantic information in unstructured text data, making the cognitive level evaluation inefficient. Therefore, this study proposed the bidirectional gated recurrent convolutional neural network combined with an attention mechanism (AM-BiGRU-CNN) deep neural network cognitive level evaluation method, and based on Bloom’s taxonomy of cognition objectives, taking the unstructured interactive text data released by 9167 learners in the massive open online course (MOOC) forum as an empirical study to support the method. The study found that the AM-BiGRU-CNN method has the best evaluation effect, with the overall accuracy of the evaluation of the six cognitive levels reaching 84.21%, of which the F1-Score at the *creating* level is 91.77%. The experimental results show that the deep neural network method can effectively identify the cognitive features implicit in the text and can be better applied to the automatic evaluation of the cognitive level of online learners. This study provides a technical reference for the evaluation of the cognitive level of the students in the online learning environment, and automatic evaluation in the realization of personalized learning strategies, teaching intervention, and resources recommended have higher application value.

## Introduction

Compared to traditional classroom teaching, online learning breaks the traditional teaching form and provides learners with abundant learning resources, diversified learning methods, and an accessible learning space, making learners the learning leaders. However, it requires that learners have a clearer understanding of the individual and the environment, to be able to clarify their learning demands and cognitive level, and reasonably adjust the learning strategies, to achieve the goal of online learning (Li et al., 2017). Learning process evaluation helps learners find out the problems and deficiencies in their online learning process in time, thereby guiding and improving online learning strategies, optimizing the learning experience, and promoting more effective online learning. The cognitive level of learners during online learning is an essential indicator for evaluating the effect of online learning. A timely evaluation of the cognitive level of learners helps them understand their cognitive level and adjust learning strategies in time (Feng et al., 2016). It can also help teachers obtain learners’ cognitive level information in time, implement teaching strategies more accurately, and provide personalized teaching interventions.

Massive open online courses (MOOCs) allow numerous people worldwide to access the knowledge they otherwise would not have online. Unlike traditional classrooms, the primary way for students and teachers to interact is through MOOC discussion forums, which encourage students to think critically, expand their knowledge horizons, and deepen their understanding of themes. Many researchers have studied MOOC forum discussion posts. For example, Stump et al. (2013) introduced a classification framework for developing and testing MOOC forum posts, categorizing many posts into a manageable number of categories, to carry out further analysis in the target area of interest. Chaturvedi et al. (2014) proposed three machine learning models to automatically classify MOOC forum discussion posts to help teachers get timely feedback and design intervention measures as needed. Chandrasekaran et al. (2015) marked a large MOOC forum corpus to enable supervised machine learning methods to automatically identify interventions that promote learning and prompt teachers when and how to intervene in discussions. Arguello and Shaffer (2015) automatically categorized the speech act categories of MOOC forum discussion posts (questions, answers), helping teachers intervene with learners by answering questions, solving problems, and providing feedback at appropriate times. Wang et al. (2015) classified MOOC forum discussion posts by content analysis and explored the relationship between students’ cognitive behaviors such as enthusiasm, constructiveness, and interaction and their learning outcomes by establishing a linear regression model. Bakharia (2016) used machine learning algorithms to classify the confusion, urgency, and sentiment of MOOC forum posts and explored the performance of different classifiers in cross-domain classification, emphasizing the necessity of transfer learning and domain adaptive algorithms. These studies have laid the foundation for the classification research of MOOC forum discussion posts, but few studies have evaluated learners’ cognitive level based on MOOC discussion posts.

Currently, there has been some research on the evaluation of learners’ cognitive level. For the problem of the level of learners’ cognitive level, the most influential is Bloom’s taxonomy of cognitive objectives (Bloom et al., 1956). With the development of education and teaching, some researchers have revised it. In the revised edition, cognition is divided into two dimensions. The cognitive process dimension is divided into six levels from low to high: *remembering*, *understanding*, *applying*, *analyzing*, *evaluating*, and *creating* (Anderson et al., 2001). For the method of cognitive level evaluation, there are mainly content analysis methods (Henri, 1992; Zhou and Han, 2018; Zhou et al., 2018; Liu et al., 2019), learning analysis (Feng et al., 2016), and traditional machine learning methods such as support vector machine (SVM) (Hsu et al., 2011; Wang et al., 2016), naive Bayesian (Yu et al., 2012; Zhang, 2018), and decision tree (Li, 2019). However, the content analysis requires manual coding, which requires a high level of research ability of the analyst. Learning analysis technology ignores the implicit semantic information in the unstructured text data. And the traditional machine learning method belongs to the category of shallow learning, which requires manual selection of many data features, is time-consuming and labor-intensive, and has poor generalization ability.

About four-fifths of the data in an organization are open and unstructured, and these unstructured data are rarely used (IBM Corporation, 2019). There are many procedural interactive learning behaviors in the online learning process, and the interaction of learners is not only a static knowledge acquisition process but also a creative cognitive process (Rowntree, 1995), and the unstructured interactive data generated along with the interactive process can become the basis for practical evaluation. The language-based unstructured interactive text data in the forum area are used as the explicit form of scholars’ thinking expression and knowledge processing, containing rich semantic information and often reflecting the hidden learning state (Wang et al., 2015). These interactive text data can reflect learners’ cognitive development and learning experience more truly, which is an essential basis for identifying learners’ cognitive level and autonomous inquiry ability (Witten et al., 2011). Making full use of the unstructured interactive text data in the online learning process to automatically mine the hidden cognitive features in the text and realize the automatic evaluation of learners’ cognitive level is an urgent problem to be solved.

The gradual maturity of natural language processing (NLP) technology has made computers increasingly capable of textually processing information (Zhen and Zheng, 2020). Deep learning based on deep neural networks is generally a multi-layer network that includes an input, a hidden, and an output layer. With the continuous iterative training process, the network will fit a complex function with many parameters and continuously update the weight parameters. The multi-layer neural network fits the actual data as much as possible and learns the feature information implicit in the input data (Chen et al., 2019). Existing studies have shown that deep neural networks combined with word vector representation can be better used for feature extraction of text data (Zong et al., 2019). At present, convolutional neural networks (CNNs) (Kim, 2014), recurrent neural networks (RNNs) (Cho et al., 2014), and attention mechanisms have been widely used in text semantic feature mining tasks with good results (Gao et al., 2018; Zhou and Bian, 2019; Lyu and Liu, 2020). Based on the unstructured interactive text data in the online learning process, the interactive text data in this study refers to the discussion forum posts. This research intends to use deep learning technology to mine the cognitive information contained in the text, construct a cognitive level evaluation method for online learning, and realize automatic evaluation of learners’ cognitive level in the process of large-scale online learning, thereby providing a new technical reference for the real-time analysis and monitoring of online learning.

## Materials and Methods

**FIGURE 1:**
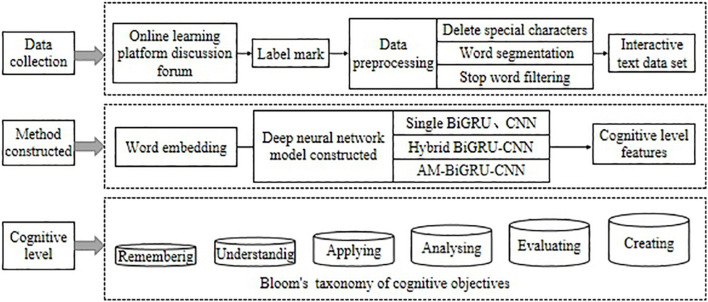
Overview of the cognitive level evaluation.

This study aims to construct a cognitive level evaluation method for online learners, which can automatically extract learners’ cognitive level information from the discussion posts posted by learners in the course forum. The constructed method will realize an accurate evaluation of learners’ cognitive level. It can help teachers understand the cognitive state of students in real-time and further personalize hierarchical teaching in real-time. The design of our study is mainly divided into three steps: data collection, method construction, and cognitive level evaluation, as shown in Figure 1.

### Data Collection

**FIGURE 2:**
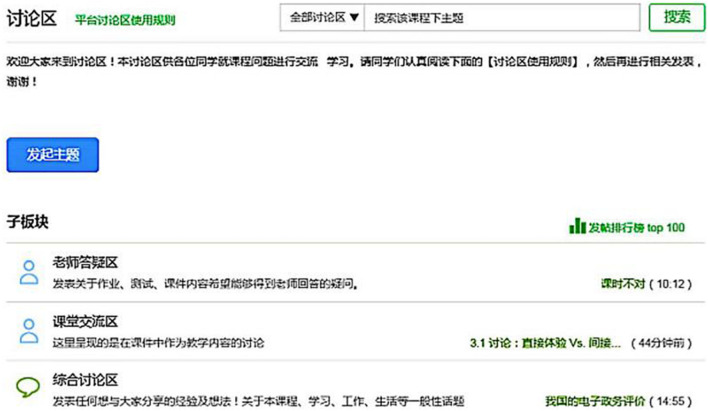
The structure of the discussion forums.

The data collection of this research comes from the open course of *Introduction to New Media*, which Zhejiang University sets on the Chinese MOOC platform. This course belongs to the National Quality Course program. A National Quality Course refers to exemplary courses with the characteristics of first-class teachers, first-class teaching content, first-class teaching methods, first-class teaching materials, and first-class teaching management (Ministry of Education of the People’s Republic of China, 2013). The content of the course is mainly to explore the interactive relationship between new media and society. On the one hand, it focuses on the shaping of new media by various social forces, and on the other hand, it discusses the impact of new media on all levels of society. From September 2014 to June 2020, the course was held 12 times, and the number of participants was about 160,000. This study collected interactive text data during the course 10 times. According to our observations, most of the learners of this course did not publish discussions in the course forum area, and many comments posted by many learners directly copied the opinions of others. This study does not include such opinions in the statistical scope. Further observations found that fewer learners published multiple original discussion posts, so this research stipulates that each learner only selects the most original discussion posts. According to the statistics of this research, 12,783 online learners participated in the interactive discussion of the original innovation. These discussion data are in the discussion area of the course. The course discussion area is the primary place for the learners to interact, and it consists of three major sections: teacher answering area, course discussion area, and comprehensive discussion area, as shown in Figure 2. The topic posts in each section have the following structure: title, content, and reply (optional). The title is an overview of the content, and the content is a detailed description of the poster. The reply is all discussion posts under the topic (the number may be 0). The topics of the teacher answering area are mainly homework, tests, and courseware content. The topics of the course discussion area are mainly about the teaching content in the courseware, and the topics of the comprehensive discussion area include lessons, learning, work, and life experience sharing. Teachers or learners initiate a discussion on a topic of interest by initiating topics.

We used a crawler program to obtain the text data and preliminarily collated and filtered the data, removed the non-cognitive discussion posts, such as “when will the electronic certificate be issued,” and finally selected the valid interactive text data published by 9167 learners. Further labeling and data preprocessing operations were performed on these data. In the labeling process, this research manually labeled the collected interactive text data as 0, 1, 2, 3, 4, and 5 categories according to Bloom’s cognitive level keywords (Chruches, 2015; Wang et al., 2020). These six categories represent the cognitive level of *remembering*, *understanding*, *applying*, *analyzing*, *evaluating*, and *creating*. For example, “认知领域的概念是什么？ (What is the concept of the public domain?),” according to the cognitive level keywords “是什么 (What is),” the text was classified as a *remembering* level, so the text was labeled with the number 0 label; “对定义中的’传播系统’应如何理解 (How to understand the ‘communication system’ in the definition?),” according to the cognitive level keywords “理解 (understand),” the text was classified as an *understanding* level, so the text was labeled with the number 1 label. The data labeling was completed by 11 people for 1 month, including 9 postgraduates and 1 undergraduate, and 1 expert professor engaged in interdisciplinary research in educational psychology and computer science. Before labeling, the experts organized all the manual labelers to conduct pre-training to have a deep understanding of Bloom’s cognitive target classification theory and the content of this online course. Ten annotators were divided into two groups during the labeling process, and five annotators formed a group. The five annotators in the same group did not have any discussion during the annotation process. If three annotators’ labels for the same text were the same, we considered the annotations valid. For controversial data texts, the expert and annotators would have a meeting to determine their cognition level uniformly. Cohen’s Kappa was used to test the inter-annotator agreement, the Kappa scores of the two groups ranged from 0.70 to 0.83 and 0.76 to 0.89, respectively, and the average Kappa scores were 0.79 and 0.82, respectively, which showed high inter-rater reliability. The description of the data set and summary statistics from the data set are shown in Table 1.

**TABLE 1:** Description of the data set and summary statistics.

	Average length	Minimum length	Maximum length	Tokens	Total number of discussion posts
Description	76	3	1261	9657	9167

### Methodology

The discussion posts in the MOOC forum generated during the online learning interaction process implied the learners’ cognitive level information. This paper proposes a bidirectional gated recurrent convolutional neural network model based on the attention mechanism (AM-BiGRU-CNN), which can extract the cognitive level features of the discussion posts to realize the automatic evaluation of the cognitive level of online learners.

The attention mechanism (Bahdanau et al., 2015) can help the network pay attention to the words that contribute more to the evaluation of the cognitive level and give them higher weight during the network training process, which is beneficial to improve the evaluation effect of the mode. Therefore, we introduced the attention mechanism at the word embedding layer. The long- and short-term memory network (LSTM) (Hochreiter and Schmidhuber, 1997) is a deep neural network with memory function, which controls the state of memory cells through the input gates and forgetting gates so that it can filter the information that input the memory in the timing input signal, and forget the useless historical information. The output gate controls the hidden state information, which contains the highly integrated feature information of input and historical information. This structure and data processing mode enable LSTM to continuously memorize and process long-term complex historical information and extract practical semantic features based on the contextual information of the discussion posts. A gated recurrent unit (GRU) (Cho et al., 2014) is a variant of LSTM, which retains the memory function of LSTM, and has a more straightforward network structure that makes training faster. However, a single GRU can only calculate the information at the next time based on the information at the last time and cannot calculate the information at the last time based on the information at the next time. The bidirectional gate recurrent unit (BiGRU) adds a reverse GRU based on the single sequential GRU, which combines the forward GRU and the reverse GRU to capture the contextual semantic information between texts (Cheng et al., 2020). Therefore, this paper uses BiGRU to better capture sentence global semantic information.

English text is composed of words, and each word is composed of several of the 26 letters. A single letter often does not represent a special meaning, and spaces initially separate the words. While Chinese text is different, a single Chinese character can express a precise meaning, the combination of characters can form words with different meanings, and the combination of words can form text information with different meanings. Due to the peculiarity of Chinese, this paper further uses a CNN (Kim, 2014) to learn the local information between Chinese text words. The CNN mainly uses convolution sliding windows to perform convolution operations to obtain n-gram feature information, such as “I love learning, but I tend to get nervous during exams, leading to bad grades,” assuming that the convolution window is 3, we can get local semantic information such as “I love learning” through the operation of convolution.

In this study, the Word2Vec model combined with the attention mechanism, BiGRU, and CNN model mentioned above construct a deep neural network method to extract cognitive features of the interactive text in the online learning platform and realize automatic evaluation of learners’ cognitive level.

#### Word Embedding

The word vector plays a vital role in NLP. It can convert the extracted online learning interactive text data into a vector representation that can be processed by a computer, thereby solving the problem of text data representation. The traditional one-hot vector cannot measure the similarity between words, and there is a problem of sparse data (Johnson and Khoshgoftaar, 2020; Sung et al., 2020). This study uses the skip-gram (continuous skip-gram) model in the Word2Vec model (Mikolov et al., 2013) to train data and learn the context of words, which can map each semantically similar word to a similar position in a low-dimensional vector space to better express the semantic information of words. This study collected 9,567 different words, including 198 unregistered words. After checking, these words have nothing to do with the cognitive evaluation of the corpus. So, the average value of all vectors is used to represent them (Zhen and Zheng, 2020).

#### Single Bidirectional Gate Recurrent Unit, Convolutional Neural Networks Model

**FIGURE 3:**
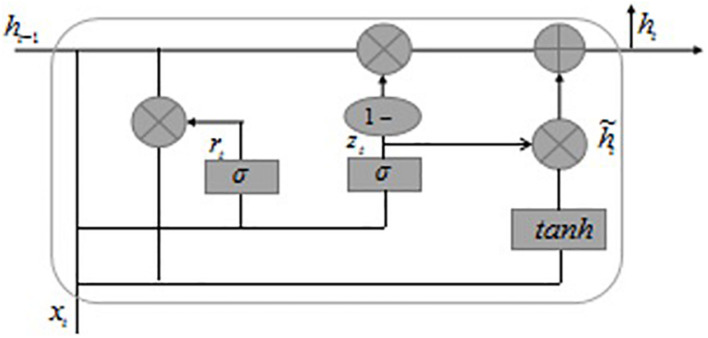
GRU model structure.

Gated recurrent unit and LSTM are both models proposed to solve long-term memory gradient disappearance and gradient explosion in the RNN network (Zhang et al., 2019). Compared with the LSTM model, GRU has a reduced “gate” structure, which is only composed of update gate *z* and reset gate *r*, to achieve fewer parameters to make the model training faster. The core of the GRU network lies in the two different “gate” mechanisms in the structure, which control the semantic information flow of the memory unit. The GRU model structure is shown in Figure 3.

The basic principle of the GRU model is shown in Eqs. 1–4 in the Supplementary Appendix. The weight values in GRU are constantly updated with the training of the network. The GRU neural unit is mainly based on the input at the previous time and the current time, through the gating unit settings of the new gate *z* and reset gate *r*, thereby controlling the update of the information in the memory unit state and ultimately retaining the text features that are more beneficial to the target task. A single GRU can only calculate the information at the next moment based on the semantic information at the last moment. The BiGRU model includes a forward GRU and a reverse GRU. The semantic information of the text is obtained from the forward and reverse directions. GRUs in each direction is connected so that the model can better focus on contextual information. The calculation of BiGRU is to concatenate the hidden layer output obtained by the forward GRU and the hidden layer output obtained by the reverse GRU.

**FIGURE 4:**
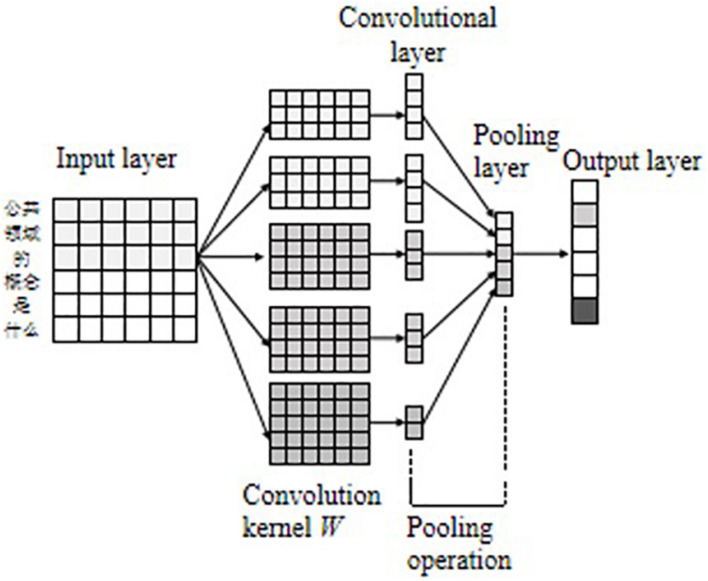
CNN model structure.

Convolutional neural networks was first applied in the field of computer vision, and in recent years has been gradually applied to NLP tasks and has achieved good processing results (Cheng et al., 2020). CNN is mainly composed of the input layer, the convolutional layer, the pooling layer, and the output layer. The convolutional layer performs feature extraction on the data passing through the input layer, and the pooling layer further filters the features extracted by the convolutional layer to select important local features. The model structure is shown in Figure 4.

Assuming that the maximum length of the input sentence in the data set is *N*, the text can be expressed as a two-dimensional matrix composed of *N* d-dimensional word vectors *x*_*i*_ ∈ *R*^*N*×*d*^. The convolutional layer uses the convolutional kernel to extract rich local features of the input matrix. For the convolutional kernel *w* ∈ *R*^*h*×*d*^, where *h* is the width of the convolutional kernel window to control the number of words, and *d* is the word vector’s dimension. After a kernel convolution operation, the local feature can be obtained, as shown in Eq. 5 in the Supplementary Appendix. When the convolution window scans the entire text, the feature map of the complete sentence can be obtained, as shown in Eq. 6 in the Supplementary Appendix.

The pooling layer further features filtering of the convolutional feature map to obtain critical local features. This study uses global maximum pooling (Zheng and Zheng, 2019) to sample the feature information, as in Eq. 7 in the Supplementary Appendix. Assuming that the number of convolution kernels *W* is *m*, then *m* convolution features *ĉ_j_* can be finally obtained, which are spliced and fused to obtain the final feature map *C*. Finally, the feature information sampled by the pooling layer is used as the input of the fully connected layer to obtain the result of the output layer, as showed in Eq. 8 in the Supplementary Appendix.

#### Hybrid Bidirectional Gate Recurrent Unit-Convolutional Neural Network Model

**FIGURE 5:**
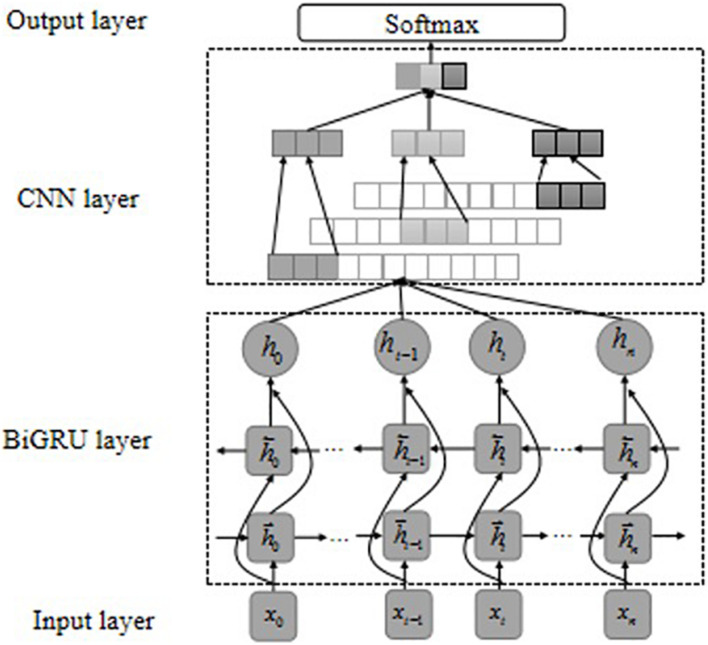
Hybrid BiGRU-CNN model structure.

Bidirectional gate recurrent unit and CNN show different advantages when representing the same text but also have some shortcomings. BiGRU is good at modeling sequence data and can establish an effective text representation through the long-term dependence of learning time features and sentences. It is successfully applied to NLP tasks, but local features of the text cannot be better extracted (Cheng et al., 2020). CNN has been proven to be able to learn most local features from natural language and has achieved good results in sentence classification (Zheng and Zheng, 2019). It uses a convolutional sliding window to obtain the most prominent features in a sentence and attempts to extract effective text representations by identifying the most influential n-gram information in different semantics. Moreover, the training speed is faster, but it is challenging to capture long-distance semantic features and ignore the contextual semantic information between texts (Xuanyuan et al., 2021). In order to make full use of the advantages of BiGRU and CNN, we combine the above two single models to construct a hybrid gated recurrent convolutional neural network (BiGRU-CNN), as shown in Figure 5. First, we converted a text containing n words into a vectorized representation *X* = *x*_1_, *x*_2_, *x*_3_, …, *x*_*n*_, *X* ∈ *R*^*n*×*d*^, and then input the vectorized representation into the BiGRU model. After calculating the BiGRU neural unit, the contextual semantic information is extracted by BiGRU and output the feature representation *H* = {*h*_1_, *h*_2_, *h*_3_, … *h*_*n*_}, *H* ∈ *R*^*n*×*k*^, where *k* is the dimension of the BiGRU hidden layer unit. Then, the obtained contextual semantic feature representation *H* is input into the CNN, and the CNN performs local feature extraction on the *H*, and finally obtains the output y after the *softmax* function.

#### AM-BiGRU-CNN Model

**FIGURE 6:**
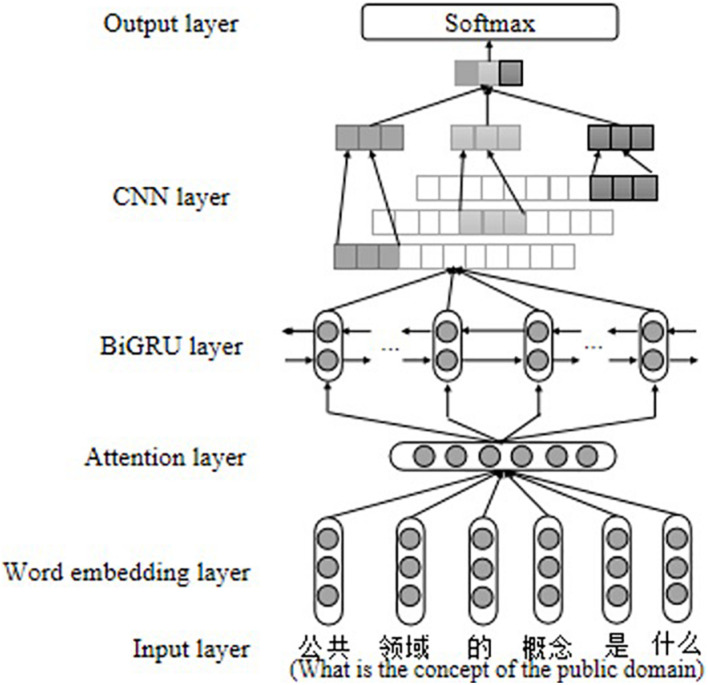
AM-BiGRU-CNN model structure.

The attention mechanism was first applied to the field of computer vision. Bahdanau et al. (2015) applied the attention mechanism to text processing for the first time and achieved good results. The sentence is composed of words, and each word has a different contribution to the final expression of the semantic information of the sentence. The attention mechanism can capture the most contributed words in the text, which helps the model obtain the semantic features of the sentence more effectively. The basic idea of the attention mechanism is explained in Eqs. 9–11 in the Supplementary Appendix. In the evaluation of the cognitive level, each word in a sentence has a different impact on the cognitive level of a sentence, especially related cognitive keywords, which can often directly reflect the cognitive level of learners. Therefore, this study adds an attention mechanism to the word embedding layer of BiGRU-CNN. Suppose a text is segmented to obtain *n* words, and each word is transformed into a vector representation through word embedding *x*_*t*_, Here *x*_*t*_ is the *h*_*i*_ in Eq. 9. First, *x*_*t*_ is activated by a *tanh* function to get the implicit representation *u*_*i*_, and then the *softmax* function is used to calculate the importance of *u*_*i*_ to get the respective weight α_*i*_s, and finally the weight of each word is multiplied with the corresponding vector representation to obtain a word representation with weight α_*i*_*x*_*i*_, which is then input into the BiGRU-CNN network to obtain AM-BiGRU-CNN, as shown in Figure 6.

#### Model Training

The goal of model training is to minimize the loss function, even if the error between the predicted value and the actual value obtained by training is minimized. This study uses the multi-class cross-entropy loss function to train the model, and the calculation method is shown in Eq. 12 in the Supplementary Appendix. In order to avoid the model from overfitting in the training model, we adopted *L*_2_ regularization (Zhou et al., 2021), which is the hyperparameter of *L*_2_ regularization.

### Automatic Evaluation of Cognitive Level

Bloom et al. (1956) proposed a taxonomy of educational objectives consisting of three domains: cognitive, effective, and psychomotor. The cognitive domain is related to thinking, knowledge acquisition, and knowledge application, and it is the most widely used and cited taxonomy in education (Ullah et al., 2019). With the development of teaching, Anderson et al. (2001) revised the original one-dimensional taxonomy of cognitive objectives to two-dimensional, including the knowledge (factual, conceptual, procedural, and metacognitive knowledge) and the cognitive process. The cognitive process is divided into six levels from lower to higher: *remembering*, *understanding*, *applying*, *analyzing*, *evaluating*, and *creating*. The specific divisions and data examples are shown in Table 2.

**TABLE 2:** Evaluation framework of cognitive level and the data examples.

Dimension	Indicators	Meaning	Interactive text data example
Cognitive level	*Remembering*	Refers to extract relevant knowledge from long-term memory	“公共领域的概念是什么？” (“What is the concept of the public domain?”) “新媒体是一个相对的概念, 是继报刊、广播、电视等传统媒体以后发展起来的新的媒体形态, 包括网络媒体…” (“New media is a relative concept, it is a new form of media that has developed after traditional media such as newspapers, radio, and television, including online media…”)
	*Understanding*	Refers to constructing meaning from teaching information disseminated verbally, written, or graphically	“对定义中的’ 传播系统’ 应如何理解？” (“How should we understand the ‘propagation system’ in the definition?”) “我理解的油门代表信息的社会需求, 刹车代表法律法规的底线” (“I understand that the throttle represents the social demand for information, and the brake represents the bottom line of laws and regulations”)
	*Applying*	Refers to the execution or use of a certain procedure in a given situation, including execution and implementation	“如何将新媒体更好的应用于教育教学中？” (“How to better apply new media to education and teaching?”) “运用短视频平台进行推广, ‘经济能支持的话会找有影响能力的人物做推广, ‘有效地将一项新媒体技术或产品进行扩散∘” (“Use the short video platform for promotion. If the economy can support it, it will find influential people to promote it, and effectively spread a new media technology or product”)
	*Analyzing*	Refers to the decomposing of a material into its constituent parts and determining the relationship between the constituent parts to form an overall structure	“感知有用性和感知易用性有什么区别？” (“What is the difference between perceived usefulness and perceived ease of use?”) “新媒体与传统媒体最主要的区别在于互动性传…” (“The main difference between new media and traditional media is interactivity…”)
	*Evaluating*	Refers to making judgments based on certain standards, including verification and judgment	“网上很多人会有网络暴力的行为, 我们每个人该如何看待这样的现象？” (“Many people on the Internet have online violence. How should each of us view this phenomenon?”) “如何看待特斯拉的“维权”呢？很多车主说∘, 他们比较在意他人的眼光, 说是特斯拉女车主的行为影响了他们的用车体验, 但是车还是好好的” (“How to view Tesla’s ‘rights protection’? Many car owners say that they are more concerned about the eyes of others. It is said that the behavior of female Tesla owners has affected their car experience, but the car is still good”)
	*Creating*	Refers to the reorganization of various elements to form a consistent or functional whole or the reorganization of elements into a new model or structure	“进入电子时代, 人们会面临被电子产品‘取代’的危险吗？” (“In the electronic age, will people face the danger of being “replaced” by electronic products?”) “新媒体会融合不同的媒介, 让人们可以通过多感官结合来获得新的信息∘” (“New media will integrate different media, allowing people to obtain new information through the combination of multiple senses”)

The revised version of Bloom’s taxonomy of cognitive objectives (Anderson et al., 2001) integrates the research results of the psychology field on cognitive psychology, which is more in line with the development of the cognitive level of student psychology and is scientific and operational in practice. It has been widely used in education research. Therefore, this study uses the revised version of Bloom’s cognitive process dimension as the final output of the cognitive level evaluation method to realize the automatic evaluation of the cognitive level.

## Results

### The Cognitive Level Distribution of Online Learners in the Online Course *Introduction to New Media*

**FIGURE 7:**
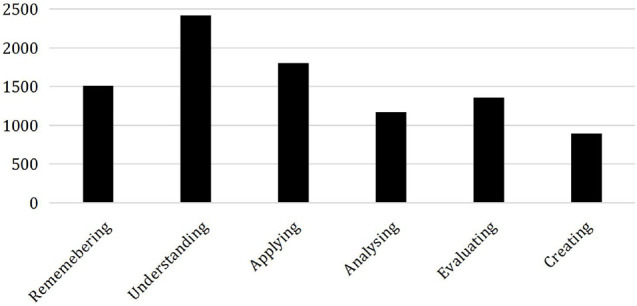
Distribution of cognitive level.

According to the collection of interactive text data in the first step, the interactive content published by 9167 online learners of the online course *Introduction to New Media* was preprocessed and labeled according to Bloom’s cognitive level keywords (Chruches, 2015; Wang et al., 2020). After we complete the label annotations of all discussion posts, we separately count the number of discussion posts belonging to these six cognitive levels. The distribution of cognition at each level is shown in Figure 6. The number of learners whose cognitive level is at the six levels of *remembering*, *understanding*, *applying*, *analyzing*, *evaluating*, and *creating* is 1,512, 2,419, 1,806, 1,174, 1,359, and 897, respectively. The distribution of the labeled data set at the six cognitive levels is shown in Figure 7. It can be found that the learner’s cognitive level at the *understanding* level is the most, accounting for about 26.39% of the total number, followed by learners at the *applying* level, and the learner’s cognitive level at the *creating* level is the least, accounting for about 9.79%.

### Automatic Evaluation Result of the Cognitive Level

In order to better extract the cognitive information from the discussion posts text, the data need to be preprocessed, including removing punctuation, word segmentation, and removing stop words. First, we removed the punctuation contained in the text, such as particular characters, spaces, and punctuation marks, to eliminate the noise in the text data. Then, word segmentation operations were performed on the text. We used the precise mode in the Jieba word segmentation library to complete word segmentation. Moreover, the stop word operation was finally performed. For example, “this,” “it,” and other functional words have no actual meaning, they should be removed. During model training, the data set is sampled and processed to balance the data at each level, and the data are divided into a training set and a test set according to a 4:1 ratio. The model is fully trained on the training set and then automatically evaluates the text data’s cognitive level on the test set. In order to verify the effects of the four deep neural network methods constructed above, this study uses four evaluation indicators: precision, recall, F1-score, and accuracy as the evaluation standard of the model. The specific calculation equation is shown in Eqs. 13–16 in the Supplementary Appendix.

The setting of hyperparameters during the experiment is very critical to the effect of deep neural network model training. The main parameters and corresponding parameter values in the experiment of this study are shown in Table 3.

**TABLE 3:** Hyperparameter settings of this study.

Hyperparameter	Set value
Maximum sentence length	256
Dimension of word vector	300
BiGRU hidden layer size	64
Convolution kernel window size	3
Number of convolution kernels	256
Batch size	64
Learning rate	0.01
Stop early	15
L_2_ regularity coefficient	0.001
Optimization algorithm	Adam

According to the above evaluation indicators and method parameter settings, the results of various indicators obtained through experiments are shown in Table 4.

**TABLE 4:** Experimental results of cognitive level evaluation.

Model	Metric	Cognitive level							
		Remembering	Understanding	Applying	Analyzing	Evaluating	Creating	Micro avg.	Macro avg.
BERT	Precision	70.24	65.68	82.93	87.57	77.89	75.56	76.71	76.55
	Recall	68.12	71.43	77.37	49.33	90.57	96.53	76.71	75.56
	F1-score	69.17	68.43	80.06	63.11	83.75	84.76	76.71	74.88
	Accuracy	76.41							
CNN	Precision	69.88	65.68	82.99	77.64	84.90	86.83	78.31	77.96
	Recall	71.18	69.81	77.31	78.29	84.27	89.77	78.31	78.44
	F1-score	70.30	67.40	79.93	76.07	82.23	88.19	78.31	78.01
	Accuracy	78.13							
GRU	Precision	70.26	67.34	77.84	74.19	83.84	87.35	77.07	76.82
	Recall	73.88	73.74	80.35	76.41	77.47	79.24	77.07	76.85
	F1-score	72.21	70.45	79.10	75.29	80.53	83.10	77.07	76.74
	Accuracy	77.07							
BiGRU	Precision	68.83	70.49	79.67	76.25	82.25	89.78	78.21	77.88
	Recall	77.66	68.81	80.34	76.51	80.44	83.57	78.21	77.89
	F1-score	72.98	70.56	80.00	76.38	81.34	86.57	78.21	77.82
	Accuracy	78.21							
BiGRU-CNN	Precision	75.74	71.69	81.88	73.60	87.13	89.32	80.28	79.89
	Recall	71.82	78.64	80.54	80.00	82.32	88.77	80.28	80.35
	F1-score	73.64	74.61	81.07	76.50	84.63	88.99	80.28	79.91
	Accuracy	80.28							
AM-CNN	Precision	72.65	76.03	86.28	74.12	88.79	91.07	82.02	81.49
	Recall	79.19	75.13	79.88	84.00	83.84	89.54	82.02	81.93
	F1-score	75.76	75.52	82.87	78.71	86.15	90.21	82.02	81.54
	Accuracy	82.02							
AM-GRU	Precision	76.37	78.65	80.72	76.19	88.13	91.32	82.11	81.90
	Recall	76.80	73.92	86.68	82.43	84.20	61.87	82.11	81.99
	F1-score	76.51	76.15	83.59	79.13	86.09	89.61	82.11	81.92
	Accuracy	82.11							
AM-BiGRU-CNN	Precision	79.24	77.96	75.72	76.20	90.01	93.85	84.21	83.78
	Recall	76.34	80.77	87.01	87.06	84.17	91.10	84.21	84.06
	F1-score	77.70	79.34	86.21	81.24	86.96	91.77	84.21	83.87
	Accuracy	84.21							

From Table 4, among the single CNN, GRU, and BiGRU neural network methods, the single GRU model has the lowest accuracy, while the BiGRU model has the best evaluation effect. The overall accuracy of the six cognitive levels is 78.21%. The accuracy of the CNN model is 1.06% higher than that of the GRU model, but it is 0.08% lower than that of the BiGRU model. Moreover, it can be found that the F1-Score of the GRU model is relatively low, while the F1-Score of the BiGRU model reaches the highest level of *remembering*, *understanding*, *applying*, and *analyzing*, and the F1-score of the CNN model reaches the highest level of *evaluating* and *creating*. It shows that the single BiGRU and CNN models have their merits in different feature extraction capabilities. From the experimental results of the hybrid BiGRU-CNN model, it can be found that combining the BiGRU and CNN models can further increase the accuracy of the model to 80.28%, which is 2.07 and 2.15% higher than the accuracy of the single BiGRU and CNN models, respectively. Observing the F1-score, we can find that the F1-score of the model at this time achieves the optimal effect on the 6 cognitive levels. The F1-score at the levels of *remembering*, *understanding*, *applying*, and *analyzing* is 0.66, 4.05, 1.07, and 0.12% higher than the BiGRU model, respectively. In order to verify the effect of the attention mechanism, this study has added the attention mechanism to the single CNN, GRU models, and the BiGRU-CNN model, respectively. It can be found that the accuracy of the AM-CNN, AM-GRU, and AM-BiGRU-CNN models are all significantly higher than the accuracy of the model without an attention mechanism. Compared with the single CNN and GRU models, the accuracy of AM-CNN and AM-GRU models is 3.89 and 5.04% higher, respectively. The AM-BiGRU-CNN hybrid model with the attention mechanism has reached the highest accuracy of this model, and it is 84.213%. Whether it is based on a single model or a hybrid BiGRU-CNN method, the attention mechanism can enable the method to achieve a higher accuracy rate, which verifies the effectiveness of the attention mechanism for this method. At the same time, it can be observed that the F1-score of the AM-BiGRU-CNN method on the six cognitive levels of interactive text is higher than that of the other six models. Compared with the CNN model, the F1-score is increased by 7.40, 11.94, 6.28, 5.17, 4.73, and 3.58%, respectively. Compared with the BiGRU model, it is increased by 4.72, 8.78, 6.21, 4.86, 5.62, and 5.20%, respectively, compared with the BiGRU-CNN model, it is increased by 4.06, 4.73, 5.14, 4.74, 2.33, and 2.78%. In addition, we compared the pre-trained Bert model with the model proposed in this paper and found that the accuracy of the AM-BiGRU-CNN model is much higher than that of the Bert model. Many experiments have shown that this Bert model can be used to achieve an excellent performance for various NLP sub-tasks. However, this does not mean that this network is perfect. The premise for the Bert model to achieve extremely high accuracy is the support of big data, which means the demand for data scale and hardware. Although many scholars have made lightweight improvement work, training-related networks still require high hardware configuration and plenty of time (Xuanyuan et al., 2021). Our experiments also show that RNN and CNN series networks are still the higher priority choices in lightweight requirements on small- and medium-sized data sets.

### Visualization of AM-BiGRU-CNN Evaluation Effect

**FIGURE 8:**
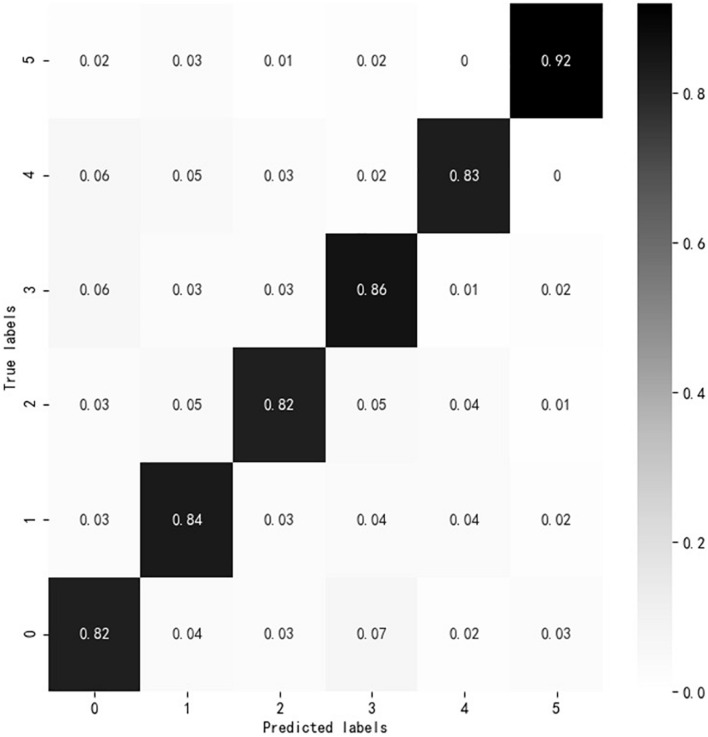
Confusion matrix analysis results.

According to the experiment results, the overall evaluation effect of AM-BiGRU-CNN is the best. In this study, a visual analysis of the evaluation effect of the AM-BiGRU-CNN method is performed, as shown in Figure 8. The figure shows the analysis result of the normalized confusion matrix of AM-BiGRU-CNN. The vertical axis (True labels) represents the actual cognitive level of the text, and the horizontal axis (Predicted labels) represents the evaluated cognitive level of the text by the method. The numbers 0–5 on the axis represent the online learning interactive texts of six cognitive levels: *remembering*, *understanding*, *applying*, *analyzing*, *evaluating*, and *creating*. The value in the matrix represents the recall evaluated by the method on the cognitive level text. The greater the value of recall, the darker the color. It can be found that the value on the diagonal is the largest, indicating that the cognitive level evaluated by this method is consistent with the actual cognitive level in most of the texts, which verifies the effectiveness of the evaluation method.

**FIGURE 9:**
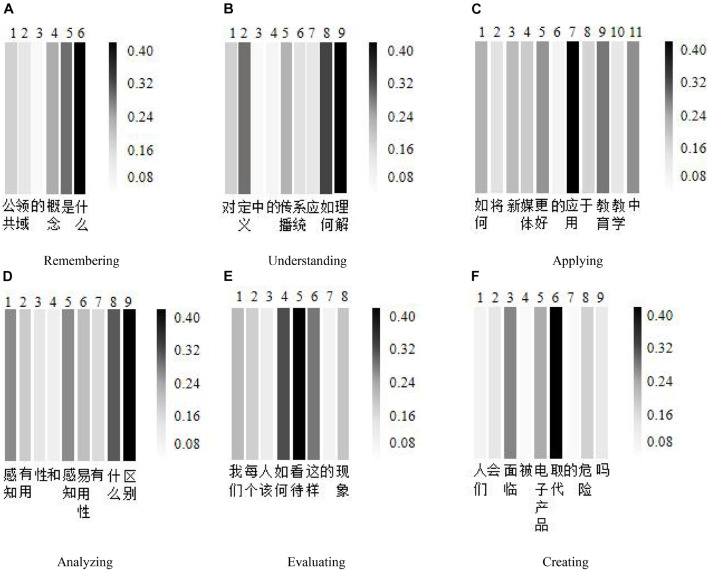
Heat map of attention mechanism weight. **(A)** Remembering. **(B)** Understanding. **(C)** Applying. **(D)** Analyzing. **(E)** Evaluating. **(F)** Creating.

In order to visually show the effect of the attention mechanism in the AM-BiGRU-CNN method, this study uses the matplotlib library to visualize the distribution of attention weights in the experiment. Based on the data set of this study, an interactive text is selected from each cognitive level as an example for the experiment. *Remembering*: “公共领域的概念是什么 (what is the concept of the public domain),” *understanding*: “对定义中的传播系统应如何理解 (how to understand the communication system in the definition),” *applying*: “如何将新媒体更好的应用于教育教学中 (how to better apply new media to education and teaching),” *analyzing*: “感知有用性和感知易用性有什么区别 (what is the difference between perceived usefulness and perceived ease of use),” *evaluating*: “我们每个人该如何看待这样的现象 (how should each of us view such a phenomenon),” and *creating*: “人们会面临被电子产品取代的危险吗 (will people be in danger of being replaced by electronic products).” For each of the above cognitive level texts, the Jieba word segmentation tool is used to segment the text. Take the cognitive level text of *remembering* and *understanding* as an example, respectively. The original sentence becomes six words {公共 领域 的 概念 是 什么} and nine words {对 定义 中 的 传播 系统 应 如何 理解} after word segmentation. In the same way, the same word segmentation is performed on the other four cognitive levels, and the corresponding 11, 9, 8, and 9 words are obtained, respectively. Based on these words, the attention weight heat map is drawn as shown in Figure 8. The larger the gray value in the figure, the higher the distribution of the attention weight value and the greater the importance of the word to the evaluation of the cognitive level. From Figures 9A–F, we can see that the model assigns high weights to the “是 (is)” and “什么 (what)” of the cognitive level of *remembering*, the “如何 (how)” and “理解 (understanding)” of the cognitive level of *understanding*, the “应用 (applied)” of the cognitive level of *applying*, the words “什么 (what)” and “区别 (distinguishment)” of the cognitive level of *analyzing*, the “如何 (how)” and “看待 (view)” of the cognitive level of *evaluating*, and the “取代 (replace)” of the cognitive level of *creating.* These words are all closely related to the cognitive keywords of the corresponding cognitive level. This result shows that the word attention mechanism can effectively identify words that significantly impact the cognitive level evaluation results.

In addition, according to the attention mechanism, we obtained a series of words with high attention weight at each cognitive level. We express it in English in Table 5.

**TABLE 5:** Words with high attention.

Cognitive level	Words
*Remembering*	Define (定义), describe (描述), find (寻找), identify (识别), locate (定位), list (列举), outline (概述), point to (指向), state (陈述), study (学习), what (什么), when (何时), where (哪里), which (哪一个), who (谁), refer (提到), duplicate (重复), memorize (记得), name (命名), mention (提及), record (记录), recall (记起), repeat (重复), reproduce (再现), Baidu (百度)
*Understanding*	Compare (比较), conclude (总结), contrast (对比), demonstrate (表现), explain (解释), interpret (解释), paraphrase (解释), match (匹配), retell (转述), summarize (总结), understand (理解), illustrate (表明), classify (划分), convert (转换), defend (辩护), discuss (讨论), express (表达), distinguish (区分), give example(s) (举例), include (包含), relate (关联) indicate (表明), review (见解), select (选择), translate (翻译), for example (例如)
*Applying*	Adapt (改编), determine (判断), develop (扩展), draw (绘制), illustrate (绘制), apply (应用), modify (改编), organize (组织), practice (实践), present (表达), produce (生产), select (挑选), show (表达), sketch (绘制), solve (解决), respond (回应), use (使用), compute (计算), change (修改), choose (选购), discover (发现), employ (采用), manipulate (操作), operate (操作), prepare (配备), extend (推广), schedule (进度), model (建模), how to use (如何用)
*Analyzing*	Analyze (分析), contrast (对比), correlate (相关), diagram (图解), differentiate (区分), examine (观察), explain (解释), group (分组), observe (观察), reason (推断), review (复审), sequence (排序), sort (排序), survey (调查), categorize (归类), compare (比较), separate (分割), deconstruct (分解), compute (计算), distinguish (区别), illustrate (说明), interpret (解释), ask (询问), indicate (指出), belong (属于)
*Evaluating*	Assess (评估), choose (选择), conclude (总结), consider (权衡), critique (批评), determine (决定), estimate (估计), evaluate (评估), interpret (解释), justify (证明), prove (证明), recommend (建议), summarize (总结), support (支持), test (测试), verify (验证), appraise (评价), discriminate (鉴别), value (价值), detect (检测)
*Creating*	Arrange (安排), combine (结合), compose (组合), coordinate (协调), create (创造), design (设计), develop (开发), formulate (制定), generate (生成), imagine (构想), interact (交互), invent (发明), portray (描写), produce (创作), publish (发表), rearrange (重组), refine (提炼), replace (取代), reorganize (重组), revise (修改), rewrite (改写), synthesize (合成), write (写作), hypothesize (设想), assemble (组合), devise (设计), plan (计划), reconstruct (重建)

## Discussion

This study first analyses the cognitive level distribution of the overall interactive text data in the online course *Introduction to New Media*. Then, based on the experimental data, the automatic evaluation results of the cognitive level of the four deep neural networks on the course discussion data are discussed. Finally, the enlightenment of the study results in the process of teaching and learning is discussed.

### The Cognitive Level of Learners Is Different, and the Overall Cognition Level Is Not High Enough

According to Figure 6, it can be seen that the discussion content published by the learners of this online course during the learning process is distributed at six different cognitive levels, and different learners have different cognitive levels. Overall, there are more interactive forums at the lower cognitive level of *understanding* and fewer interactive forums at the higher level of *creating*. It should be emphasized that a low level of cognition does not necessarily mean that the learner’s learning results are not ideal, because the online course selected in this article is *Introduction to New Media*, which mainly allows students to understand new media. It teaches students factual knowledge, so the cognitive level of students rarely reaches the *creating* level.

### The Deep Neural Network Method Can Effectively and Automatically Evaluate the Cognitive Level Contained in Online Discussion Forums

According to the experimental results in Table 3, among the four deep neural network methods, the evaluation effect of the BiGRU method is better than that of CNN. This is because BiGRU regards the text as time-series information, considering the influence of the previous text on the subsequent text, and the influence of the subsequent text on the previous text, to better extract the contextual semantic information. BiGRU-CNN can effectively extract the contextual global semantic information of the discussion forums and focus on the essential semantic information locally so that the overall accuracy rate is more accurate than the evaluation of two single methods. The AM-BiGRU-CNN method has the best evaluation effect because the attention mechanism is added to the embedding layer, making the method pay attention to the cognitive keywords in the text to evaluate the cognitive level implied in the discussion forums more accurately. The evaluation effect of all these methods on *remembering*, *understanding*, and *analyzing* texts is lower than that of the other three cognitive levels texts. Because some of the cognitive level keywords contained in the different cognitive level text are relatively similar, and the interactive text presents a certain degree of crossover in cognitive semantic features, it is not easy to distinguish them accurately. According to the visual analysis results of Figures 7, 8, the AM-BiGRU-CNN method can effectively automatically evaluate the cognitive level implied in the online discussion forum, and the attention mechanism can focus on the words that are more important to the cognitive level evaluation in the text and give them higher attention weight. These results illustrate the effectiveness of adding the attention mechanism to the deep neural network cognitive level evaluation method. At present, the MOOC platform can basically realize the automatic management of online learning, which can facilitate teachers to manage courses better, but it still cannot meet the needs of learners according to the individual characteristics and learning conditions of each learner. In the actual platform development and improvement process, the AM-BiGRU-CNN cognitive level automatic evaluation model proposed by this research can be embedded into the platform to provide students or teachers with automatic cognitive level evaluation functions to help teachers in real time master the cognitive status of each student. It can also allow students to monitor their current cognitive level, to effectively use metacognitive skills to properly adjust the cognitive process, thereby achieving successful online learning.

### The Enlightenment of the Automatic Evaluation Method of Cognitive Level in the Teaching and Learning Process

Online learning is currently one of the important ways for learners to acquire knowledge. Different learners have different cognitive levels in the learning process. Automatic evaluation of learners’ cognitive levels is the basis for monitoring and evaluating the effect of large-scale online learning. It is also a prerequisite for improving learners’ online learning effects by providing personalized learning strategies or personalized learning support. From the perspective of learners, real-time grasping of their cognitive level helps them position themselves to formulate their learning plans and learning strategies and adjust the plan and strategies according to the changes of their cognitive level during the learning process to develop learners’ metacognitive ability further. For example, in this online course, learners whose cognitive level is at a low level of *remembering* or *understanding* can pay attention to change their learning attitudes in daily learning, recognize their dominant position during the learning process, clarify the purpose of learning, cultivate the desire for knowledge, and apply the learned knowledge to practice. For learners at intermediate cognitive levels, such as *applying* or *analyzing*, they can actively ask the teacher more questions during the process of online learning, stimulate their creative thinking in the interaction with the teachers, and exercise their high-level cognitive ability. For learners whose cognitive level is at a higher level of *evaluating* or *creating*, they can try to normalize higher-order thinking to maintain higher-order thinking skills during other learning tasks. From the perspective of teachers, teachers can only rely on their personal experience to subjectively judge the process performance of learners in practice. However, in a large-scale online learning environment, this empirical and subjective evaluation will be challenging, and teachers cannot know about everyone simultaneously. The real-time automatic evaluation of learners’ cognitive level can help teachers quickly comprehend the cognitive level of each learner. Different teaching strategies can be developed for learners with different cognitive levels. For example, teachers can divide learners into different levels according to their cognitive level and provide different hints and guidance to learners at different levels to carry out hierarchical teaching to achieve the purpose of personalized learning. From the perspective of learning platforms, automatic evaluation of learner’s cognitive level is helpful to realize personalized recommendation of learning resources. Therefore, it has important significance and value to construct an efficient learner’s cognitive level evaluation method based on the interactive text data of the online learning platform for improving the effectiveness of online learning and achieving personalized teaching.

## Conclusion

Real-time evaluation of learners’ cognitive level in online learning helps to monitor learners’ own cognitive state to adjust learning strategies to improve the quality of online learning. In this study, interactive text data of learners were taken from the online learning platform and preprocessed, the automatic cognitive evaluation methods for BiGRU, CNN, BiGRU-CNN, and AM-BiGRU-CNN deep neural network were constructed. The case analysis of the online course *Introduction to New Media* on the Chinese MOOC was carried out. The experimental results show that the deep neural network can realize the automatic evaluation of learners’ cognitive level based on Bloom’s taxonomy of cognitive objectives. The accuracy of the hybrid AM-BiGRU-CNN model constructed in this paper reached 84.21%, the evaluation accuracy was better than its sub-models constituting the hybrid model and better than the Bert model based on pre-training. Unlike ordinary text classification tasks, Bloom’s taxonomy of cognitive objectives has a certain degree of overlap and ambiguity in the semantics of each cognitive level, which makes it more difficult for the model to evaluate different cognitive levels accurately. Referring to the current emotion multi-classification tasks with similar characteristics, in Lin et al.’s (2019) three emotion classification tasks, the highest F1-score of each category is only 64.38%. In our study, in the cognitive six-layered task, the F1-score of each level was above 75%, and the highest reached 91.77%. To a certain extent, it shows that the AM-BiGRU-CNN model proposed in this paper can effectively evaluate the cognitive level of learners in real-time based on Bloom’s cognitive target classification theory. The cognitive level automatic evaluation model constructed in this study makes up for the shortcomings of traditional manual coding and traditional machine learning methods in cognitive level evaluation and provides a technical reference for student cognitive level evaluation and automated evaluation in a large-scale online learning environment. It is of great significance for the realization of personalized online learning.

Nevertheless, this study has several limitations. The adaptability of the model domain proposed in this paper needs to be improved. This paper evaluates learners’ cognitive level based on a supervised deep learning model. The training data set was used from the online course discussion texts of engineering disciplines. The model is highly domain-dependent on the training data set. If the research in this article is directly applied to online courses in other fields, such as science or humanities and social sciences, learners’ interactive content or expressions may be different. The description style of the text content will also be different, often using the unique description object of the domain, professional domain words, so that the accuracy of the model on the interactive data set of other domains is not high. In addition, when constructing the cognitive level evaluation model, this paper only relies on the automatic extraction of text features by the hybrid deep neural network and does not further consider the fine-grained language features such as the syntactic rules. For example, the negative rules and the addition of negative words can make the meaning expressed in the text opposite, and a no more advanced neural network model is used, so the model’s accuracy needs to be further improved. Finally, in the process of data labeling, this article only relies on manual labeling of data, which is time-consuming and laborious, and much manual labeling of data sets will lead to more subjective data set labels.

In the future, we will consider more online courses in different fields and collect more interactive text data in different fields. Considering that manual labeling of each data set is time-consuming and laborious, we will consider using machine learning. The algorithm realizes the automatic labeling of the data set. Second, we will consider using transfer learning algorithms or domain adaptive algorithms to enhance the domain adaptability of the cognitive level evaluation model. Finally, we will consider incorporating syntactic rules to improve the accuracy of the model further.

## Data Availability Statement

The raw data supporting the conclusions of this article will be made available by the authors, without undue reservation.

## Ethics Statement

Ethical review and approval was not required for the study on human participants in accordance with the Local Legislation and Institutional Requirements. Written informed consent from the patients/participants or patients/participants legal guardian/next of kin was not required to participate in this study in accordance with the National Legislation and the Institutional Requirements.

## Author Contributions

YaC: conceptualization, methodology, thesis and whole process guidance, funding acquisition, and supervision. YiC: methodology, software, and writing – original draft preparation. HC: writing – review and editing and data curation. ZC: data analysis and investigation. GW: visualization and investigation. JH: validation. All authors listed have made a substantial, direct and intellectual contribution to the work, and approved it for publication.

## Conflict of Interest

The authors declare that the research was conducted in the absence of any commercial or financial relationships that could be construed as a potential conflict of interest.

## Publisher’s Note

All claims expressed in this article are solely those of the authors and do not necessarily represent those of their affiliated organizations, or those of the publisher, the editors and the reviewers. Any product that may be evaluated in this article, or claim that may be made by its manufacturer, is not guaranteed or endorsed by the publisher.
