# Supplementary material for: A Cognitive Level Evaluation Method Based on a Deep Neural Network for Online Learning: From a Bloom’s Taxonomy of Cognition Objectives Perspective
Source: Front Psychol. 2021 Oct 14;12:661235. doi: 10.3389/fpsyg.2021.661235 (PMC8551629; doi:10.3389/fpsyg.2021.661235)
Supplement: Supplementary file 1 [file Data_Sheet_1.pdf]

Supplementary Appendix

<table>
<tr><td>GRU</td><td>

$$r_t = \sigma(W_r x_t + U_r h_{t-1}) \quad (1)$$

$$z_t = \sigma(W_z x_t + U_z h_{t-1}) \quad (2)$$

$$\widetilde{h_t} = \tanh(W x_t + U(r_t \odot h_{t-1})) \quad (3)$$

$$h_t = (1 - z_t) h_{t-1} + z_t \widetilde{h_t} \quad (4)$$

r_t represents the reset gate, z_t represents the update gate, $\widetilde{h_t}$ represents the candidate hidden information to be updated, and h_t represents the output information of the final hidden layer obtained through the control of two "gate" mechanisms. Suppose that the current time point is t, the input at the current time point is x_t, and the input information in the entire time series is expressed as $X = \{x_1, x_2, \ldots, x_t\}$. Specifically, in a text task, X corresponds to a text record, where at each time point the input x_t represents a single word in the text after word segmentation. Then the input of the GRU model includes the input x_t at the current time and the output h_{t-1} of the hidden layer at the previous time point, and the output of the model includes the output h_t of the hidden layer at the current time point and the current memory unit state c_t.

</td></tr>
<tr><td>CNN</td><td>

$$c_{ji} = f(W_j \cdot X_{i:i+h-1} + b) \quad (5)$$

$$c_j = [c_{j1}, c_{j2}, \cdots, c_{jN}] \quad (6)$$

$$\hat{c}_j = \max\{c_j\} \quad (7)$$

$$y = softmax(W \cdot C + b) \quad (8)$$

$W_j \in R^{h \times k}$ is the weight of the j-th convolution kernel, $b \in \mathrm{R}$ is the bias, c_{ji} represents the local features obtained after convolution of word x, and c_j represents the feature map. $W \in R^d$ represents the weight of the fully connected layer, $b \in R$ is the bias, and y is the output result.

</td></tr>
<tr><td>Attention mechanism</td><td>

$$u_i = \tanh(W_i h_i + b_i) \quad (9)$$

$$\alpha_i = \frac{\exp(u_i^T u_w)}{\sum_i \exp(u_i^T u_w)} \quad (10)$$

$$h_i = \sum_i \alpha_i h_i \quad (11)$$

u_w is the randomly initialized context vector, which is updated during the training process, u_i is the result of a fully connected operation of the hidden layer vector h_i,

</td></tr>
</table>

	W_i and b_i are the weight matrix and bias term calculated, respectively, for the attention, and α_i is the attention score of i-th word in the sentence.
Cross-entropy lossfunction	$$\mathbf{J(\theta) = -\frac{1}{k}\sum_{i=1}^{k} \bar{y}_i \log(y_i) + \lambda \| \theta \|_F^2} \quad \textbf{(12)}$$<br>$\bar{y}_i$ is the predicted labels, y_i is the probability value of each type of label after softmax, and k is the number of label categories. In this study, the value of k is 6.
Evaluation indicators	$$\boldsymbol{Precision = \frac{TP}{TP+FP}} \quad \textbf{(13)}$$<br>$$\boldsymbol{Recall = \frac{TP}{TP+FN}} \quad \textbf{(14)}$$<br>$$\boldsymbol{F1 = \frac{2*Precision*Recall}{Precision+Recall}} \quad \textbf{(15)}$$<br>$$\boldsymbol{Accuracy = \frac{TP+TN}{TP+FP+TN+FN}} \quad \textbf{(16)}$$<br>*TP* is the number of positive samples that are correctly classified, *FP* is the number of positive samples that are incorrectly classified, *TN* is the number of negative samples that are correctly classified, and *FN* is the number of negative samples that are incorrectly classified. The positive and negative samples here refer to the evaluation criteria in the two-classification task to the multi-classification task. The category in each category is regarded as a positive sample, and the other categories are regarded as a negative sample.

2
